# The effect of New Zealand blackcurrant on sport performance and related biomarkers: a systematic review and meta-analysis

**DOI:** 10.1186/s12970-020-00354-9

**Published:** 2020-05-27

**Authors:** A. J. Braakhuis, V. X. Somerville, R. D. Hurst

**Affiliations:** 1 Discipline of Nutrition, Faculty of Medical & Health Sciences, The University of Auckland, Private Bag, Auckland, 92019 New Zealand; 2 The New Zealand Institute for Plant and Food Research Limited, Food Innovation Portfolio, Food & Wellness Group, Private Bag, Palmerston North, 11600 New Zealand

**Keywords:** Anthocyanin, Berry, Exercise, Athlete

## Abstract

**Background:**

Blackcurrants have come to be regarded as a superfood because of their high polyphenol content, namely anthocyanins. While many berry types have been studied, blackcurrant-anthocyanins may be the superior berry when it comes to athletic performance. The purpose of the review was to evaluate the effects of blackcurrant supplementation on athletic performance, oxidative markers, cognition, and side effects.

**Methods:**

Systematic review and meta-analysis. Review manager software (version 5.3) was used for the meta-analysis. The risks of bias was independently assessed using the guidelines and criteria outlined in the Cochrane Handbook for Systematic Reviews of Interventions. The data sources for the search included MEDLINE (Ovid), Google Scholar databases, additional references lists, conference proceedings and grey literature until August 2019. Eligibility Criteria included all blackcurrant (New Zealand derived) interventions, randomised control trials, human participants, placebo-controlled only.

**Results:**

A total of 16 separate studies met the criteria for inclusion in the systematic review, with 9 studies contributing to this sport performance meta-analysis. There was an improvement in sport performance when supplementing with blackcurrant, 0.45 (95% CI 0.09–0.81, *p* = 0.01). The effective dose appears to be between 105 and 210 mg of total blackcurrant anthocyanins, prior to exercise. There were insufficient studies reporting oxidative markers, cognitive effects or biomarkers, and/or side effects to comment on the mechanism of action.

**Conclusion:**

Blackcurrant has a small, but significant, effect on sport performance, with no known detrimental side effects.

## Introduction

The popularity of perceived naturally occurring plant extracts and phytochemicals to enhance physical performance, exercise recovery and maintain health has increased dramatically in recent years. Interest in the health properties and benefits of blackcurrants for active individuals started over 10 years ago [1, 2]; however the last 5 years has seen a surge in research interest [3]. In 2018, the International Olympic Committee (IOC) released a statement on the efficacy of dietary supplements for athletes, in which polyphenols were reported to increase mitochondrial biogenesis and endurance performance, at least in mice [4]. The IOC highlighted the importance of conducting reviews using a systematic process and in their hierarchy of scientific evidence, systematic reviews and the process of meta-analysis were considered the gold standard [4]. A semi-recent systematic review and meta-analysis investigated the effect of polyphenols, albeit a very broad dietary group, on athletic performance in humans [5] and demonstrated a significant improvement when taking polyphenols for 7 days or more. Given the review included all polyphenols and accepting the broad nature of the inclusion criteria, translating this finding into a recommendation for athletes is problematic. In the current review we investigate blackcurrants (*Ribes nigrum*), which are naturally high in a particular range of polyphenols and may provide an opportunity to be specific regarding optimal dosing strategies.

Berries are a brightly coloured fruit which have been investigated for their health-promoting effects, particularly blackcurrant, blueberries (*Vaccinium spp*), and blackberry (*Rubus spp*). Berries contain high concentrations of a particular class of flavonoids called anthocyanins, but each berry type has a specific make-up of anthocyanins [3, 6]. Anthocyanins are natural pigments responsible for the blue, purple, red and orange colours of many fruits and vegetables [6]. While the total daily consumption of total anthocyanins has been estimated to be between 3 and 215 mg/day [6], the optimal intake of total and specific anthocyanins is uncertain. In general, blackcurrant contains between 130 and 460 mg/100 g fruit weight of total anthocyanins [6] and the predominant type is delphinidin-3-rutinoside. In contrast, blueberries contain 62–300 mg/100 g fruit weight of total anthocyanins [6] with the predominant type being malvidin-3-monogalactoside [7]. Blackcurrant anthocyanins have shown to alleviate inflammation and oxidative stress, while preventing the depletion of mitochondrial content and damage [8], not reported with blueberry anthocyanins. Thus blackcurrant anthocyanins (delphinidin glycosides) appear to be a more effective antioxidant than blueberry anthocyanins (malvidin glycosides) [9, 10]. The physiological effects of the different berries suggests not all polyphenols and anthocyanins have the same bioactivity.

While the general health benefit claims of blackcurrants are warranted, there are few studies comparing the anthocyanin content of different blackcurrant varieties grown in a range of countries. New Zealand blackcurrants (NZ BC) have been reported to have higher concentrations of anthocyanins and other phytochemicals than those grown in other countries, which is a likely consequence of an environment with high UV and long, sunny days. The anthocyanin content of juice produced from NZ BC has previously been shown to be between 336 and 850 mg/100 mL, in comparison to non-NZ blackcurrants with an anthocyanin content ranging from 170 to 310 mg/100 mL of juice [11]. While acknowledging the total amount of blackcurrant anthocyanins consumed is likely to be a key factor, for pragmatic reasons a concentrated dose might be preferable for athletes, particularly prior to an event. As such, the purpose of the review was to determine whether blackcurrant anthocyanins, particularly from NZ BC, alter direct and indirect aspects of athletic performance. In particular, does NZ BC improve athletic performance and modulate oxidative and cognitive effects?

## Main text

### Methods

#### Search strategy and study selection

A search strategy was developed using appropriate MeSH terms and boolean operators, in which the predominant search terms covered blackcurrant, sport, exercise and cognition with limits for randomised control trials and healthy human participants. Interventions were required to be placebo-controlled NZ BC interventions, include a maximal exercise performance test and be written in English. Only studies conducted at sea-level or standard oxygen concentrations were included. The initial strategy was developed prior to the full investigation. An electronic search was conducted for all studies investigating the effects of blackcurrant, derived from New Zealand, on exercise performance via MEDLINE (Ovid) and Google Scholar databases. The MEDLINE search strategy can be located in the Supplementary Data files. Relevant reference lists and conference proceedings follow-up provided valuable unpublished data. The search period was not restricted by year of publication (up to August 15th 2019). The title and abstract of each study were initially screened during the electronic search to exclude irrelevant studies from the database list. Pre-specified inclusion and exclusion criteria were applied to the abstracts of remaining studies. The full texts or abridged report, if sufficient information was provided, were then assessed for eligibility and those that met inclusion criteria were critically appraised and quality checked to reach a final decision. Two authors (AB, VS) carried out the search independently, and then resolved any disagreements.

The primary outcome under investigation was sport performance. Sport performance was defined by a maximal performance test and included clear performance outcomes (typically time to fatigue or time-trial time). Secondary outcomes included inflammatory/oxidative measures (protein carbonyl [PC], malondialdehyde [MDA], ferric reducing ability of plasma [FRAP], Interleukin-1 [IL-1], Interleukin-8 [IL-8], Interleukin-10 [IL-10], Interleukin-6 [IL-6], tumour necrosis factor-alpha [TNF α]); cognitive function (validated task test, for example: Stroop, Montreal cognitive assessment), monoamine oxidase activity/concentration (MAO), and any reported side effects. Inclusion criteria specified that the studies must be randomised, placebo-controlled human interventions examining New Zealand blackcurrant. Isolated strength tests and recovery protocols were excluded. Human trials on participants with Parkinson disease were also excluded, as were those without inferential statistics (SE/SD or exact *p*-values), or appropriate methodology (exercise protocol). Ethical committee approval is not required for reviews.

#### Risk of bias assessment in included studies

The risks of bias for all included meta-analysed studies were independently assessed using the guidelines and criteria outlined in the Cochrane Handbook for Systematic Reviews of Interventions [12]. The major sources of bias we chose to assess were random sequence generation (selection bias), allocation concealment (selection bias), blinding (performance bias and detection bias), and selective reporting (reporting bias), as recommended by the Cochrane Handbook [12]. Each study was also checked for bias through the involvement of a conflict of interest by manually checking the acknowledgements and funding source of each study. The risk for each major source of bias was defined as either ‘low risk’, ‘unclear risk’, or ‘high risk’ for each included trial, and a risk of bias table was generated for each meta-analysis.

#### Data extraction

The mean, standard deviation and sample size of the sport performance outcomes for intervention and control groups were extracted. Mean performance (time (min/sec), distance (m) or power (watts)) and variance data were extracted directly from the manuscripts into excel. One study was removed as the data had been reported elsewhere [13]. Data from Willems 2016 [14] was extracted from graphical presentation. Using the Review Manager software a standardised mean performance effect and standard error were calculated for each study. Acknowledging the difficulty with pooling performance data from various exercise test types, we initially conducted the adjustments for tests to exhaustion, exercise and cycle type as described elsewhere [15]. We did however find no difference in using standardised mean difference effects and prior adjustments with mean effects in this data set. Hence, to remove the subjective nature of the manual adjustments, we chose to use the standardised mean difference approach.

Data on the inflammatory and oxidative measures were extracted for at-rest and post-exercise values. If multiple post exercise values were collected, the value closest to immediately post exercise was recorded. Measures provided as a percent change from baseline were excluded as raw or statistically adjusted data were required. In studies of cross-over design that analysed the same participant data in two ways, the most common analysis approach was reported. By way of example, one study reported MDA as protein concentration in the blood, and via the erythrocyte simulation assay [16]. With regards to MDA analysis, protein concentration is the most common method of analysis and thus data reported. In addition, when the same participant data has been collected on acute versus chronic intake, data on chronic intake has been reported here, as this was the most common testing regime. Changes in oxidative stress markers were expressed as ratios (post-exercise measure/pre-exercise measure) for intervention and control. For a marker to be presented graphically there had to be at least three studies with relevant data.

Much of the variance data was presented as mean and SEM. SEM data were converted to standard deviation for standardised reporting in this manuscript. All data sources were considered for inclusion, including abstract information from conference posters, unpublished data and published manuscripts, provided appropriate inferential data could be extracted.

Side effect events were extracted as the total number of episodes over the entire supplementation period, regardless of severity. The reported outcome includes a count score of side effects and description.

#### Statistical analysis

Outcomes with equal to or greater than four independent data points were meta-analysed.

Performance data were converted into a percent difference between the intervention and control groups. The variance data between groups was calculated using the mean and standard deviation data from each group and enteredinto the Review Manager (version 5.3, Cochrane, London, UK) calculator to derive the standard error. The mean effect and standard error was used to generate the standardised mean difference. The inverse variance statistical method was chosen to generate the study effect of NZ blackcurrant anthocyanins.

Assessment of heterogeneity between included studies was evaluated by the Higgins score (I^2^). Values of I^2^ were interpreted using the guidelines outlined in the Cochrane Handbook for Systematic Reviews of Interventions [12], where 0 to 40% might not be important; 30 to 60% may represent moderate heterogeneity; 50 to 90% may represent substantial heterogeneity, and 75 to 100% may represent considerable heterogeneity [17]. The fixed effects model was adopted when the Higgins score was below 60%; otherwise the random-effects model was used.

To interpret the magnitude of change in the oxidative stress markers, the authors calculated the baseline variability as a x/÷ factor SD, calculated by the equation below. To evaluate the magnitude of change, Cohen’s thresholds of 0.20, 0.60, and 1.20 of the factor SD were used, which represents small, moderate, and large respectively.

$$ e\sqrt{\frac{\sum \left({\left\{\ln \left(\left(\mathrm{mean}/\mathrm{SD}\right)/\mathrm{mean}\right)\right\}}^2\bullet df\right)}{\sum df}} $$

## Results

**Fig. 1:**
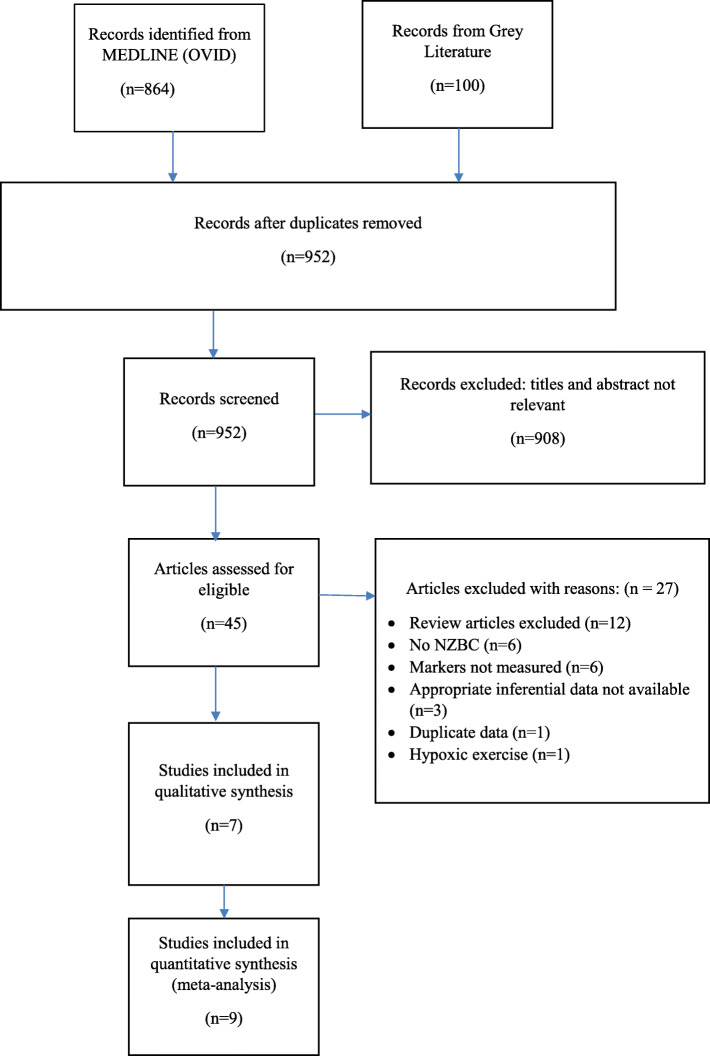
PRISMA chart outlining identification of included studies

A total of 964 articles were identified through searching databases and other sources. Following removal of duplicates, 952 articles remained. After screening by title and abstract, 45 articles remained. Following application of specific inclusion and exclusion criteria, critical appraisal, and quality checking of articles, 16 were deemed acceptable for inclusion. See Fig. 1 PRISMA Chart.

### Description of included studies

Sixteen studies were included in the review. One included sport performance, oxidative stress measures and side effects [16], eight included sport performance data only, [14, 18–24], three had oxidative stress measures [1, 25, 26], one had oxidative and cognitive measures [27], two included cognitive measures only [28, 29], and one included oxidative measures and side effects [2]. Among the included studies, six were conducted in New Zealand, the remaining in the United Kingdom. Of the nine performance studies with included performance data, all used a cross-over design, and report the effect of New Zealand blackcurrant on either cycling, running or climbing hang time.

### Meta-analysis results

#### Blackcurrant and its effect on performance

**Fig. 2:**
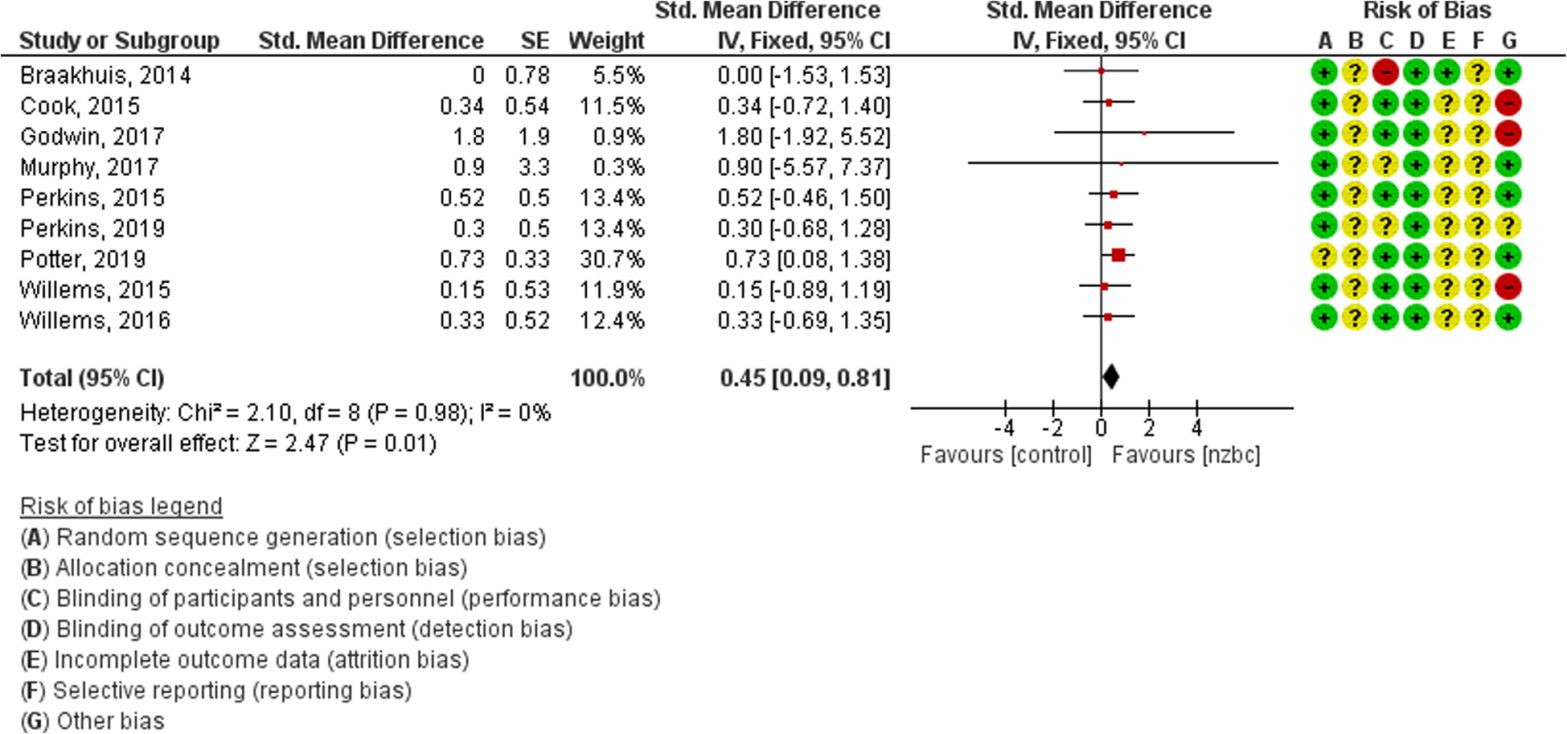
Forest plot of performance effects of NZ BC compared to placebo. Results are expressed as standardized mean differences, and 95% confidence intervals (CI)

The performance effect of NZ BC vs. placebo during exercise was investigated in nine studies. The meta-analysis using the fixed-effects model calculated the standardised mean percent effect of NZ BC on performance to be 0.45 (95% CI 0.09–0.81, *p* = 0.01), which is significant. The heterogeneity among studies included in the meta-analysis did not significantly affect the outcome (see Fig. 2).

#### Risk of bias in included studies

Whilst there are some gaps in the reported methods of included studies, there is an overall low to unclear risk that the true performance effect of NZ BC has been influenced by bias from included studies. The classification of bias is presented alongside Fig. 2. In summary, the nine studies were all classified as low risk bias for ‘random sequence generation’ as the cross over design was appropriate and randomisation was conducted. ‘Allocation concealment’ was unclear as the authors did not state their respective methods. Similarly the nine studies were assigned unclear risk bias of ‘incomplete outcome data’ as they were crossover and only included results of participants who had completed both arms of the trial. One study reported drop-out rates with reason, and rated low risk for incomplete data. All studies were classified as low risk of bias for ‘blinding’ as they were stated double-blind and mentioned intervention and control capsules were identical. A search for the associate protocol of each study was completed, however no protocol was found for all included studies. The studies were therefore all classified as unclear ‘selective reporting’ bias.

**Fig. 3:**
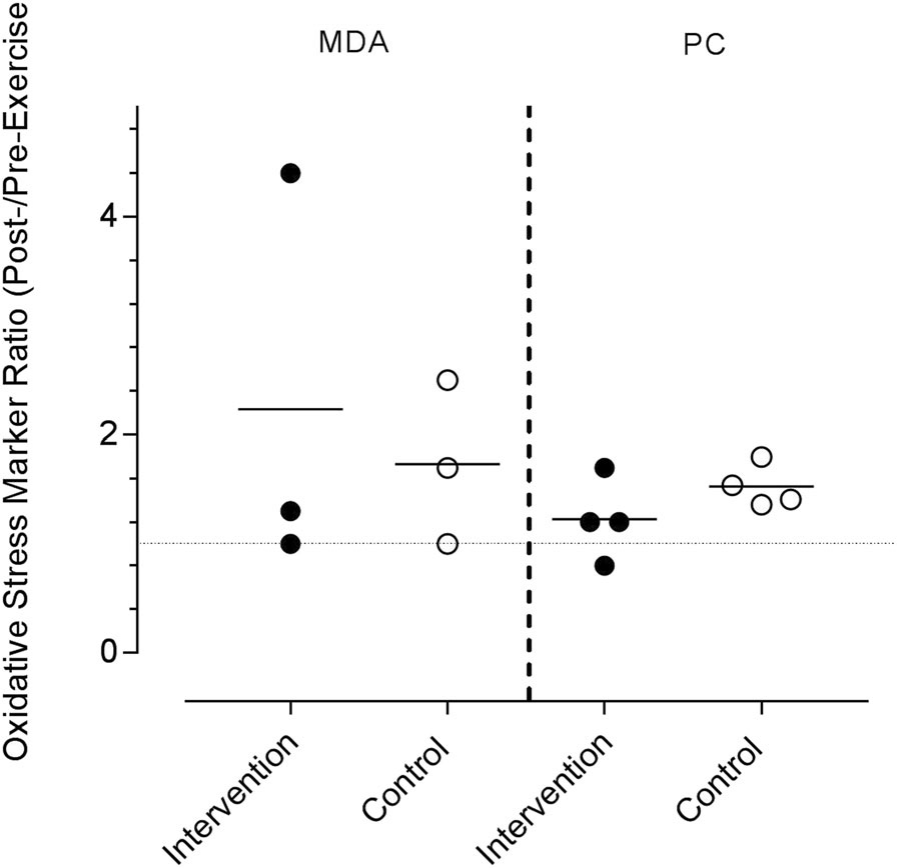
Scatter plot of the studies that investigated oxidative stress markers before and after exercise, expressed as a post-:pre-exercise ratio. The horizontal dotted line represents no change compared with baseline. The solid horizontal line represents the geometric mean. MDA, malondialdehyde; PC, protein carbonyl

The distinction in ‘other’ bias classification resulted from inadequate conflict of interest statements. Five studies [14, 16, 19, 20, 23] were classified as low ‘other’ bias as the respective authors specified the study funder had no input in study design, results and/or choice to publish (See Fig. 3). The reviewers classified the remaining studies [18, 21, 22, 24] as unclear or high risk as they provided limited conflict of interest statement regarding trial design and choice to publish by the funder. Industry funded projects were deemed high risk in other sources of bias. Missing data was deemed unclear as data on drop-outs was only available in one study. Other sources of bias include inappropriate wash-out periods which was not detected.

#### Oxidative stress biomarkers

Outcomes from MDA and PC conducted during exercise trials were graphed and generally indicate a reduction in oxidative stress under the conditions of exercise. One study did report a greater increase in MDA with the intervention than the control; the other two studies indicating reductions. Both MDA and PC were raised after exercise with x/÷ factor SDs of 2.58 and 3.36 respectively. The qualitative statement indicating the magnitude of change in the geometric mean were varied between moderate and large; however the overall change in the intervention group was similar to the control group. There were insufficient papers to graphically represent any other oxidative stress or inflammatory markers.

One additional study measured MDA and PC without an exercise trial and reported trivial differences in PC when taking NZ BC (PC, BC 0.2 vs PL 0.18 nmol/mg protein, at baseline; BC 0.13 vs 0.11 nmol/mg protein, after 12 weeks supplementation), and lower MDA when taking NZ BC (MDA, BC 17.3 vs PL 23.5 ng/mL, at baseline; BC 7 vs PL 10 ng/mL after 12 weeks supplementation) [1].

Outcome measures have been reported for TNFα, IL-6, IL-10 and FRAP pre and post-exercise in those taking NZ BC or placebo [25, 26]. The pre-exercise inflammatory markers were higher with the intervention treatment than placebo (TNFα, BC 3 vs PL 2.1 pg/mL; IL-6, BC 12 vs 10 pg/mL and IL-10, BC 80 vs 60 pg/mL) and oxidative stress marker higher on the intervention than placebo (FRAP, BC 0.15 vs 0.14%), although none of these reached significance. The post-exercise inflammatory markers were the same or lower with the intervention treatment than placebo (TNFα, BC 9 vs PL 10 pg/mL; IL-6, BC 20 vs 21 pg/mL and IL-10, BC 60 vs 60 pg/mL) and oxidative stress lower on the intervention than placebo post exercise (FRAP, BC 0.12 vs PL 0.13%).

#### Cognitive biomarkers and effects of blackcurrant

Three studies reported platelet MAO-B concentrations after consuming NZ BC or placebo. All three show clear reduction with NZ BC (Platelet MAO-B, BC 1.6 vs PL 22.1 nM H_2_0_2_ production/μg protein/min [27]; Platelet MAO-B, BC − 280 vs PL 10 nmol H_2_0_2_ change from baseline [28]; Platelet MAO-B, BC 1417.86 vs PL 1739.99 nmol H_2_0_2_ [29]). Data were reported using heterogeneous methods thus making graphing or meta-analysis problematic.

Cognitive questionnaire data were reported by one study [28] with a Stroop cognitive test accuracy (F = 0.014, *p* > 0.01) and reaction time (F = 0.33, *p* > 0.1) no better with NZ BC. However, Rapid Visual Information Processing accuracy improved (F = 5.88, *p* = 0.005) with NZ BC.

#### Reported side effects

Three of sixteen studies made specific mention of reported side effects from taking dietary supplements or drinks containing NZ BC [2, 16, 27]. One reported minor gastrointestinal upset in two of the 24 participants consuming NZ BC [16], which provided the blackcurrant intervention as a drink from a combination of powder extract and concentrated syrup. In two of the studies there were no adverse/ side effects, stomach issues or other consequences from the intervention product [2, 27]. It is worth noting that data on side effects is not systematically collected and reported in all research, we also can’t be sure the side effects reported are the direct result of NZ BC or other ingredients or products.

## Discussion

A 1% difference in athletic performance is relevant to athletes and of sufficient magnitude to affect medal rankings in an Olympic-level competition, with the potential to elevate a medal from fourth to a podium position [30]. The aim of the current review was to systematically evaluate the literature and pool the available evidence to determine the potential benefit of NZ BC on athletic performance and whether this is sufficiently relevant to matter. The meta-analysis shows a significant improvement in performance (effect of 0.45) which is quantitatively deemed a small, significant improvement. Interestingly, the magnitude of the effect we report for NZ BC is not dissimilar to caffeine (effect of 0.41 (95% CI: 0.15–0.68), *p* = 0.002) [30], which has long been touted as the most potent legal ergogenic food or supplement available.

Whilst acknowledging the majority of the performance research included in the meta-analysis were conducted on sub-elite athletes, one article conducted a sub-analysis on participants based on training load [16]. The athletes with higher training loads had a greater response to NZ BC compared with the low training load group, which might suggest both sub- and elite athletes could benefit.

A recent narrative review highlighted the physiological mechanisms behind the potential of NZ BC [3] stating acute intake may influence cardiovascular alterations such as vasodilation and increased peripheral blood flow, but longer intake durations may be required to result in changes in cellular signaling and mitochondrial adaptations. Of the nine studies included in the performance data, eight used supplement protocols of 7 days, the one remaining study supplemented for 3 weeks, suggesting 7 days is a sufficient length of time to be effective. Alongside the consideration of the ideal anthocyanin supplement length of time is the effective daily dose, which in the performance studies is generally 105–210 mg daily, and in the oxidative and cognitive inclusive studies ranges from 20 to 500 mg daily. One of the performance studies provided participants a daily dose of 300 mg of anthocyanins, and reported minimal performance effects and some minor gastrointestinal side effects, suggesting the ideal dose should be less than 300 mg daily [16]. Recent studies have investigated the dose response of NZ BC, indicating a minimum of 120 mg of blackcurrant anthocyanin taken acutely reduces oxidative stress [25].

The bioavailability of polyphenols and anthocyanins are generally thought to be poorly absorbed with 5–10% occurring in the small intestine with key metabolites reaching the bloodstream between 0.5 to 1.5 h after consumption [31], and peak anthocyanin levels reaching the blood stream 2 h post consumption [25]. As such, the timing of anthocyanin intake to the exercise is a consideration in defining an optimal consumption. Of all included studies, 7 report providing the final dose 2-h before the relevant testing took place, 2 report 3-h, 4 report ≥1-h, the remaining did not report when the participants consumed the final dose.

The summary of included studies outlined in Table 1 indicate that an NZ BC anthocyanin intake of 105–210 mg taken for 7 days with the last dose 1–2 h before activity to be most effective for performance gains. With regards to the management of exercise-induced oxidative stress, data from Table 2 demonstrate effects after a single intake of NZ BC with the minimum dose required of 120 mg of blackcurrant anthocyanins. Further studies are required to determine whether performance benefits are achieved after a single intake.

**Table 1:** Details of blackcurrant studies included in the performance meta-analysis

Study	Subjects; design	BC anthocyanin dose^**a**^	Final dose	Performance Protocol
Braakhuis, 2014 [17]	23 females (active); crossover	300 mg.d^− 1^ BC anthocyanins for 3-wk	2–3 h before test	Run time during a 5 km run time trial (25 min)
Cook, 2015 [19]	14 males (active); crossover	105 mg.d^− 1^ BC anthocyanins for 7-d	2 h before test	Cycle time during a 16.1 km cycle time trial (28 min)
Godwin, 2017 [20]	24 males (active); crossover	210 mg.d^− 1^ BC anthocyanins for 7-d	2 h before test	Sprint time during a repeated run sprint interval test to fatigue (average of sprint 3–6, 22 s)
Murphy, 2017 [21]	10 males (active); crossover	105 mg.d^− 1^ BC anthocyanins for 7-d	2 h before test	Cycle time during a twice repeated 4-km cycle time trial (12 min)
Perkins, 2015 [22]	13 males (active); crossover	105 mg.d^− 1^ BC anthocyanins for 7-d	3 h before test	Distance covered during a repeated sprint test to fatigue (4 km)
Perkins, 2019 [23]	16 males (active); crossover	210 mg.d^−1^ BC anthocyanins for 7-d	Not reported	Distance covered during a repeated sprint test to fatigue (4.7 km)
Potter, 2020 [23]	18 males, 2 females (active); crossover	210 mg.d^− 1^ BC anthocyanins for 7-d	Not reported	Hang time during a simulated rock climbing test to fatigue (30 s)
Willems, 2015 [25]	8 males, 5 females (active); crossover	138.6 mg.d^−1^ BC anthocyanins for 7-d	2–3 h before test	Power output at set lactate level during a cycle test (225 watts)
Willems, 2016 [15]	13 males (active); crossover	105 mg.d^− 1^ BC anthocyanins for 7-d	3 h before test	Run time during a run test to fatigue following a sprint test (14 min)

Key: ^a^*BC*=Blackcurrant

**Table 2:** Details of blackcurrant studies included which report oxidative, inflammatory, and cognitive outcomes

Study	Subjects; design	BC anthocyanin dose^**a**^	Final dose	Outcomes measures^**b**^
Hurst, 2019 [27]	32 males and females; parallel design	0.8, 1.6, or 3.2 mg.kg^− 1^ body weight BC extract (34% anthocyanins) (8 individuals per group). ~ 20, 40 and 80 mg BC anthocyanins consumed acutely	1 h before test	Oxidative measures (FRAP, PC)
Hurst, 2019 [28]	20 males and females; parallel design	3.2 mg.kg^− 1^ body weight BC extract (34% anthocyanins).d^− 1^ for 5-wk. ~ 80 mg BC anthocyanins daily.	2 h before test	Oxidative measures (MDA, FRAP, IL-6, IL-10, TNFα)
Lomiwes, 2019 [27]	15 males and 25 females; parallel design	4.8 mg.kg^− 1^ body weight BC concentrate diluted in water (200 mL) prepared and onsumed acutely. ~ 120 mg BC anthocyanins.	1 h before test	Cognitive measures (MAO-B). Oxidative measures (MDA)
Lyall, 2009 [2]	5 males and 5 females; crossover	240 mg BC anthocyanins consumed acutely	30-min before test	Oxidative measures (PC), Side effects
McGhie, 2007 [1]	20 male and female older adults in control and blackcurrant arm; parallel design	500 mg BC anthocyanins daily for 24 weeks from BC extract and concentrate diluted in water (200 mL)	Not reported	Oxidative measures (PC, MDA)
Watson, 2015 [30]	36 males and females, 3-arm; crossover	~ 500 mg.60 kg^− 1^ body weight BC anthocyanins extract; OR ~ 400 mg.60 kg^− 1^ body weight BC anthocyanins juice, consumed acutely	1 h before test	Cognitive measures (MAO-B, Stroop test, Digit Divigilance test)
Watson, 2018 [31]	8 males; crossover	~ 6.2 mg.kg^− 1^ body weight BC anthocyanins juice, consumed acutely	Not reported	Cognitive measures (MAO-B)

Key: a BC=Blackcurrant; b FRAP=Ferric reducing ability of plasma, PC=Protein carbonyl, MDA = Malondialdehyde, MAO-B=Platelet monoamine oxidase-B activity

The NZ BC was provided in various forms which varied between studies, including juice concentrate, powdered juice concentrate, powdered whole fruit and powdered extracted anthocyanins. One study investigated both juice (‘BlackAdder’ blackcurrant cultivar) and powdered extracted anthocyanins, reporting slightly more anthocyanin content in the powder when made up to an equivalent 200 mL serve [28]. The improvements in cognition were slightly better with the powdered extract than juice, which also had a lower blood glucose response. The predominant form of NZ BC used in studies was powdered product in capsules to help reduce placebo bias and maintain a consistent anthocyanin content.

To provide insight into the mechanistic action of NZ BC, we investigated the oxidative and inflammatory markers when taking NZ BC. The traditional approach to interpreting oxidative stress and inflammatory markers are to define a reduction as “good”, suggesting the underlying oxidative stress and inflammation are “bad.” However, it has been identified that oxidative stress can initiate signaling to support improved training adaptations and nutrients with very high antioxidant capacity may not be in the best interest of the athlete if taken chronically [32]. In the analysis, we attempted to define whether NZ BC altered the oxidative or inflammatory response to exercise and failed to see a consistent response outside the inherent variability of the biomarkers (demonstrated as the factor standard deviation presented in the results to support Fig. 3). However, of significant interest are the studies including an acute and chronic component, clearly showing an increase in inflammatory markers (TNFα, IL-6, IL-10) and an oxidative stress marker (FRAP) while ingesting NZ BC prior to exercise, but not after. It is possible the NZ BC is priming the antioxidant and inflammatory responses. NZ BC anthocyanins appear to interact with cellular antioxidant systems and mediate enhancement of antioxidant defenses and mitochondrial adaptation which are likely to be far more important than the oxidative scavenging activity [33]. Oxidative stressors that are proposed to induce mitochondrial adaptation include exercise, calorie restriction, and polyphenols acting as pro-oxidant primers [34]. The central signaling molecule likely to be important in these adaptive responses is nuclear factor (erythroid-derived 2)-like 2, also known as Nrf-2 [25]. Nrf-2 is a transcription factor and is regarded as a master regulator of antioxidant, inflammatory and mitochondrial responses. The Nrf family activate genes that encode mitochondrial respiratory chain and translocation proteins [35]. Under normal homeostatic conditions, Nrf-2 is suppressed and becomes active upon exposure to oxidative stress whereby it translocates into the nucleus and binds to the antioxidant response element of the genes of antioxidant enzymes. This results in the initiation of the transcription of the genes that control adaptive enzymes which in turn manage the oxidative stress [25, 33]. While the data we extracted in this review failed to see a difference in oxidative markers when taking NZ BC compared with control, we do acknowledge the number of studies included was very low, too low to meta-analyse or make meaningful conclusions. However, in theory, the provision of a moderate dose of BC anthocyanins may be priming the antioxidant and inflammatory processes, while activating mitochondrial growth to support peak performance.

The oxidative and inflammatory outcome measures included in this review are widely studied, but it should be acknowledged that more sophisticated measures may better differentiate changes with dietary intervention. Modern approaches include in vitro assays, metabolic and microRNA approaches, which require more research before results can be used to guide evidence-based practice. One of the limitations of the current review is the reliance on relatively crude oxidative and inflammatory markers which may in part explain the lack of clarity in the oxidative outcome measures.

Aside from the performance response, the cognitive and mood attributes appear trivial, with improvements in cognitive test score and monoamine oxidase (MAO) inhibitory effects thought to enhance cognition and mood [27, 28]. Studies have reported monoamine oxidase enzyme inhibition in humans after taking blackcurrant which may maintain neurotransmitter function, leading to improvements in cognition and mood [27, 28], and as such is a biomarker of cognition. Cognitive effects of polyphenols may result from the vasodilator activity which aligns with the short time frame of action [28]. Our results did not support a cognitive improvement.

To our knowledge, no single study has investigated nutrient supplementation and timing over a competitive season, let alone investigated the personalised approach of NZ BC. Certainly for athletes competing multiple times in a year, thought should be given to an ideal, personalised, supplement regime.

## Conclusions

New Zealand grown blackcurrants mediate a small, significant improvement on athletic performance, particularly when consumed for 7 days at a dose of 105–210 mg anthocyanins, with a final dose 1–2 h before exercise. The predominant form of NZ BC used in studies was powdered product in capsules. While the evidence for a direct effect on sport performance was clear, the mechanism is not. We found only a small number of studies that have investigated oxidative, cognitive and adverse outcomes, with no known detrimental side effects reported.

## Supplementary information

**Additional file 1.** Medline Search Strategy.

## Data Availability

Data used in this review have been sourced from available data and can be shared by contacting the primary author. The search strategy is available as supplementary information.

## Abbreviations

NZ BC: New Zealand blackcurrant
PC: Protein carbonyl
MDA: Malondialdehyde
FRAP: Ferric reducing ability of plasma
IL-1: interleukin-1
IL-8: Interleukin-8
IL-10: Interleukin-10
IL-6: Interleukin-6
TNF α: Tumour necrosis factor-alpha
MAO: Monoamine oxidase activity/concentration
