# Correction to: The effect of New Zealand blackcurrant on sport performance and related biomarkers: a systematic review and meta-analysis

**DOI:** 10.1186/s12970-020-00398-x

**Published:** 2021-01-12

**Authors:** A. J. Braakhuis, V. X. Somerville, R. D. Hurst

**Affiliations:** 1 Discipline of Nutrition, Faculty of Medical & Health Sciences, The University of Auckland, Private Bag, Auckland, 92019 New Zealand; 2 The New Zealand Institute for Plant and Food Research Limited, Food Innovation Portfolio, Food & Wellness Group, Private Bag, Palmerston North, 11600 New Zealand

**Correction to: Journal of the International Society of Sports Nutrition 17, 25 (2020)**

**https://doi.org/10.1186/s12970-020-00354-9**

The original article contains numerous errors [1] of which the corrections are detailed below:

- A further review questioned whether the authors should include a further publication, Willems et al 2019 [2]. The authors highlight that in the early additions of the manuscript Willems et al 2019 [2] was included, however one of the original reviewers stated the trial was conducted under non-normal oxygen conditions and as such should be excluded from the pooled analysis. On this basis, we justified the exclusion of the study in the methods, “Only studies conducted at sea-level or standard oxygen concentrations were included” and stand by our rationale. In the interest of full transparency, if we were to include Willems, 2019, [2] the overall effect on performance is calculated to be 0.41% (0.07-0.75).
- Concerning the meta-analysis of pooled data, the authors report a 0.45% improvement in performance with the use of New Zealand blackcurrant. In the manuscript, authors compared these results with previously published caffeine data, stating caffeine improved performance by 0.41% [3]. The comparison between our blackcurrant data and that on caffeine has been questioned. Authors have subsequently extracted the caffeine data from the reference and meta-analysed the outcomes using the exact same methodology presented in our manuscript, which included a preload adjustment for Wiles, 1992 [4] and Doherty, 2004 [5]. This new analysis, presented as a percent performance effect, showed caffeine improved performance by 1.08% (CI 0.92-1.24). Therefore, we have misrepresented the caffeine data in our original manuscript and the correct improved performance value is 1.08% (and not 0.41% as originally stated).
- While the manuscript did address general bias inherent in included studies (including heterogeneity, selective reporting and research design), authors did not specifically address publication bias, as the pooled data included less than 10 studies and such we deemed would be too speculative [6]. However, in the interest of full transparency, a post publication assessment of the funnel plot (standard error versus standard mean/mean difference) of the performance, malondialdehyde and cognitive biomarker data was visually inspected which we would tentatively interpret as symmetric, acknowledging the issues mentioned.
- Another issue raised by a fresh review was the use of the term “powdered product in a capsule” which we define broadly as a powdered fruit product, incorporating a powdered extract, a powdered concentrate or other powdered blackcurrant product as produced by the New Zealand industry. The authors focused the discussion on the active dose of anthocyanins, which we firmly believe is appropriate, as in practice the active dose could be readily achieved via any New Zealand blackcurrant powdered product and hence we stand by our use of the term “powdered product in a capsule”. In general, powdered product was a generic term to cover all commercial forms of New Zealand blackcurrant fruit powders used in these studies, rather than being a term chosen to reflect any commercial drivers.
- The authors have taken this opportunity to correct reference errors in the manuscript, as follows:
  - ○ Table 1: Potter, 2020 should read Potter, 2018 [7].
  - ○ Figure 2: Potter, 2019 should read Potter, 2018.
- During the process of publication, there was a change in the Competing Interests status of one author (Hurst) which led to a fresh review of the manuscript. The revised competing interests section is as follows:
  - ○ Braakhuis and Somerville are associated with academic institutions, with no competing interests. Hurst has dual associations with commercial organizations that may benefit financially from the sale of sports performance products made from New Zealand blackcurrants: as Principal Scientist for the New Zealand Institute for Plant and Food Research Limited: and as Chief Science Advisor for 2before Performance Nutrition Limited. Funding for this research was provided by The New Zealand Institute for Plant and Food Research. The results are presented clearly, honestly, without fabrication, falsification, or inappropriate data manipulation.

The authors appreciate the additional review and feedback initiated by the change in the competing interests of one author. The authors apologise for the misinterpretations of caffeine performance data and for any inconvenience caused.
