# Supplementary material for: The effect of New Zealand blackcurrant on sport performance and related biomarkers: a systematic review and meta-analysis
Source: J Int Soc Sports Nutr. 2020 May 27;17:25. doi: 10.1186/s12970-020-00354-9 (PMC7251677; doi:10.1186/s12970-020-00354-9)
Supplement: Supplementary file 1 — Additional file 1. Medline Search Strategy. [file 12970_2020_354_MOESM1_ESM.docx]

**Medline Search Strategy**

1. exp Plants, Medicinal/
2. Exp Ribes
3. Black currant. ti,ab.
4. Anthocyanin. ti.ab.
5. Or/1-4
6. (Athlet* or physical) adj3 performance
7. Sports.ti.ab
8. Exp Exercise
9. Exp Physical Fitness
10. (cognit* adj3 (func* or declin* or reduc* or impair* or improve* or deficit* or progress* or perform* or abilit*)).ti,ab.
11. "mental perform*".ti,ab.
12. memory.ti,ab.
13. "executive function*".ti,ab.
14. Executive Function/
15. Attention/
16. (speed adj2 processing).ti,ab.
17. "episodic memory".ti,ab.
18. Memory, Episodic/
19. Or/6-18
20. randomized controlled trial.pt.
21. controlled clinical trial.pt.
22. randomized.ab.
23. placebo.ab.
24. drug therapy.fs.
25. randomly.ab.
26. Trial.ab.
27. groups.ab.
28. or/20-28
29. exp Animals/ not humans.sh.
30. 5 and 19 and 28 and 29
